# Adjunctive non-pharmacological interventions for motor symptoms in Parkinson’s disease: a scoping review of randomized controlled trials

**DOI:** 10.3389/fneur.2026.1926421

**Published:** 2026-09-18

**Authors:** Ziyuan Wang, Jie Ruan, Mengjiao Zhang, Chen Li

**Affiliations:** 1 First Teaching Hospital of Tianjin University of Traditional Chinese Medicine, Tianjin, China; 2 National Clinical Research Center for Chinese Medicine, Tianjin, China

**Keywords:** motor symptoms, non-pharmacological interventions, Parkinson’s disease, rehabilitation, scoping review

## Abstract

**Background:**

Motor symptoms in Parkinson’s disease (PD), particularly gait, balance, freezing, and axial impairment, can remain troublesome despite optimized dopaminergic treatment. The literature on non-pharmacological adjuncts is diverse, and the label “complementary and alternative therapy” is used inconsistently across conventional rehabilitation, mind–body practices, acupuncture, cueing, and neuromodulation.

**Objective:**

To map randomized evidence on adjunctive non-pharmacological interventions for PD motor symptoms, while distinguishing complementary/integrative practices from conventional rehabilitation and other adjunctive technologies.

**Methods:**

We conducted a PRISMA-ScR-guided scoping review of PubMed and Web of Science from 1 January 2015 to 7 September 2025. English- or Chinese-language randomized controlled, randomized crossover, pilot, or feasibility trials were eligible when at least one prespecified motor or motor-function outcome was reported. Published reports and independent trials were counted separately. Non-motor outcomes were not synthesized unless they were ancillary to an otherwise eligible motor-focused trial.

**Results:**

After a study-level and report-level audit, 50 eligible reports representing 48 independent randomized trials were retained. Seven reports evaluated rehabilitation or technology-assisted training, 15 mind–body or exercise-based approaches, eight acupuncture-related or manual approaches, and 20 neuromodulation, cueing, biofeedback, or other physical interventions. Study size, intervention dose, comparator choice, motor phenotype, outcome selection, follow-up, and safety reporting varied substantially. Two pairs of publications were identified as reports from the same trial families.

**Conclusion:**

The randomized literature is broad but methodologically heterogeneous. This scoping review maps where motor outcomes have been studied; it does not establish comparative efficacy or justify treatment recommendations. Future trials and evidence syntheses should distinguish intervention classes more precisely, link multiple reports from the same trial, prespecify motor phenotypes and core outcomes, and report adverse events and adherence consistently.

**Systematic review registration:**

https://osf.io/tu5nd/, DOI: 10.17605/OSF.IO/UKH9G.

## 1 Introduction

Parkinson’s disease (PD) is a progressive neurodegenerative disorder with motor and non-motor manifestations. Bradykinesia, rigidity, tremor, postural instability, freezing of gait, and impaired balance can restrict activities of daily living and are closely related to falls and loss of independence (1, 2). Dopaminergic therapy remains central to symptomatic management, but postural control, gait initiation, and some axial deficits may respond incompletely or variably, particularly as disease complexity increases (3–5).

Interest in non-pharmacological adjuncts has therefore expanded. A conceptual problem, however, is that “complementary,” “alternative,” “integrative,” “rehabilitative,” and “non-pharmacological” are not interchangeable terms. Complementary care refers to non-mainstream practices used alongside conventional medicine, whereas alternative care implies use in place of conventional treatment; in PD, published complementary-therapy reviews commonly include acupuncture, Tai Chi, Qigong, yoga, and related mind–body or manual practices (6). Conventional physiotherapy, task-specific rehabilitation, virtual-reality training, cueing, and experimental neuromodulation do not automatically become complementary or alternative simply because they are non-drug interventions.

To avoid conflating these categories, the present revision uses the umbrella term “adjunctive non-pharmacological interventions” (ANPIs). Within that umbrella, complementary/integrative practices are described separately from conventional rehabilitation and from adjunctive technologies such as cueing, biofeedback, and non-invasive stimulation. None of the included interventions is treated as a substitute for established pharmacological or multidisciplinary neurological care.

A scoping design was selected because interventions, comparators, treatment doses, and motor endpoints were too heterogeneous for a single clinically coherent pooled effect. The objective was to map randomized evidence published from 2015 to 2025, identify which motor phenotypes and outcomes have been studied, distinguish published reports from independent trials, and describe methodological gaps that should inform future research rather than generate treatment recommendations.

## 2 Methods

### 2.1 Design and reporting framework

This scoping review was prepared in accordance with PRISMA-ScR and established methodological guidance for scoping reviews (7–10). The review was designed as an evidence map; no meta-analysis was planned because the interventions and outcome constructs were not sufficiently homogeneous.

### 2.2 Protocol registration and deviations

The project has an OSF record.^1^ The registered project initially focused on non-motor symptoms of PD. After completion of the original literature search, the review focus and title were changed to motor symptoms. The registered plan also differed from the implemented review in the breadth of eligible study designs and in database coverage. These were substantive post-registration deviations rather than minor editorial amendments. The present manuscript therefore reports the implemented methods in full and treats the OSF record as a historical record of protocol evolution, not as evidence that the final motor-focused review was prospectively prespecified. The change in outcome focus, the restriction to randomized designs, the final information sources, and the clarified language criterion are summarized in Supplementary Appendix B. This deviation is also acknowledged as a limitation in the Discussion.

### 2.3 Eligibility criteria and operational classification of interventions

Eligible participants had a clinical diagnosis of PD. Eligible reports were randomized controlled trials, randomized crossover trials, or randomized pilot/feasibility trials published between 1 January 2015 and 7 September 2025. Quasi-randomized studies were excluded. Reports had to provide at least one motor or motor-function endpoint, including UPDRS-III or MDS-UPDRS III, gait or spatiotemporal walking measures, balance, Timed Up and Go, freezing of gait, fall-related motor outcomes, tremor, hypomimia/facial motor performance, or closely related functional motor measures. Reports focused exclusively on constipation, dysphagia, cognition, emotion recognition, biomarkers, mood, or quality of life were not eligible for the motor evidence map. When an eligible motor-focused trial also reported non-motor secondary outcomes, those outcomes were not used to characterize motor efficacy.

The intervention had to be non-pharmacological and non-surgical and be used as an adjunct to, or comparison with, routine PD care. For classification, complementary/integrative practices comprised non-mainstream approaches used alongside conventional care, such as acupuncture/acupressure, Tai Chi, Qigong, yoga, meditative movement, and osteopathic/manual approaches. Conventional physiotherapy, task-specific exercise, telerehabilitation, and virtual-reality rehabilitation were classified as rehabilitation rather than CATs. Cueing, biofeedback, and non-invasive stimulation were classified as other adjunctive non-pharmacological technologies. The term “alternative therapy” was not used to imply replacement of standard PD treatment.

Comparators could include usual care, waiting list/no additional treatment, sham/placebo procedures, conventional rehabilitation, exercise, or another active intervention. Full-text reports in English or Chinese were eligible; reports in other languages were excluded. This replaces the previous ambiguous wording that English-language reports were “prioritized.”

### 2.4 Information sources and search strategy

PubMed and Web of Science Core Collection were searched for records published from 1 January 2015 through 7 September 2025; the final search was run on 7 September 2025. Search concepts covered PD, randomized trials, and a deliberately broad set of complementary, rehabilitative, exercise, cueing, virtual-reality, biofeedback, manual, and non-invasive stimulation terms. Reference lists of included reports were screened for additional eligible records. The complete database-specific search strings, field tags, date restrictions, and language limits are provided in Supplementary Appendix A.

### 2.5 Record management, study selection, and data extraction

Search results were imported into EndNote. Duplicate detection was performed using EndNote’s duplicate-matching function and was followed by manual verification based on title, authors, year, journal, and DOI/PMID where available. Two reviewers independently screened titles/abstracts and full texts and independently extracted study characteristics; disagreements were resolved by discussion. Extracted fields included publication, randomized sample size, sex distribution when reported, motor phenotype or target, intervention, comparator, treatment dose/duration, motor outcomes, follow-up, and adverse-event reporting. Missing information was coded as not reported (NR) rather than inferred.

Because more than one publication can arise from a single randomized cohort, reports suspected to belong to the same trial family were linked using author overlap, sample size, intervention/comparator structure, recruitment details, and trial characteristics. We therefore report both the number of eligible published reports and the number of independent randomized trials. In the revised tables, linked reports are flagged rather than counted as independent trials.

### 2.6 Data synthesis and limits of inference

Results were synthesized descriptively by intervention class. Formal risk-of-bias assessment and meta-analysis were not undertaken because this was a scoping evidence map rather than an efficacy review. Accordingly, the synthesis does not grade certainty, pool effect estimates, or make comparative or patient-level treatment recommendations. Between-group findings are described only when needed to clarify the nature of the evidence, and non-motor secondary outcomes are not used to support motor-effect conclusions. Safety reporting is mapped as reported or NR; absence of reporting is not interpreted as absence of harm.

## 3 Results

### 3.1 Study selection and reconciliation of reports with independent trials

The database searches yielded 351 records (PubMed, *n* = 202; Web of Science, *n* = 149). After removal of 30 duplicate records, 321 records were screened. Two hundred thirty-eight records were excluded at title/abstract screening, and 83 full-text reports were assessed. Following the revision audit, 33 full-text reports were excluded. This total includes the 30 reports excluded in the original screening plus three records removed during the present audit: one quasi-randomized report and two reports without an eligible motor or motor-function endpoint. Fifty eligible published reports remained. Two pairs of publications represented the same underlying randomized cohorts, yielding 48 independent randomized trials (Figure 1).

**Figure 1:**
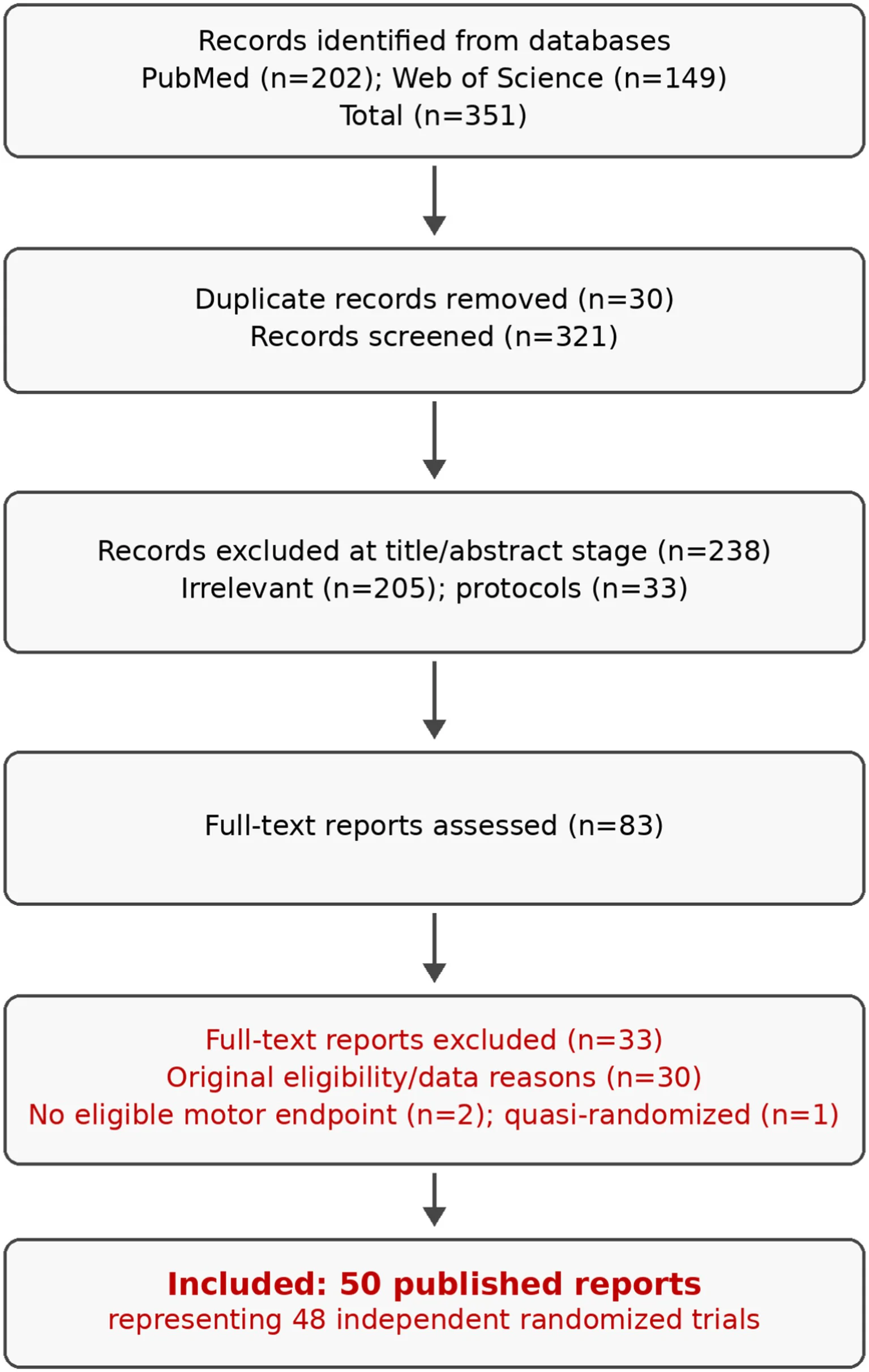
PRISMA-ScR flow diagram after report-level and trial-family reconciliation.

### 3.2 Overview of the evidence map

The 50 eligible reports were distributed across four descriptive categories: rehabilitation or technology-assisted training (7 reports), mind–body or exercise-based interventions (15 reports), acupuncture-related or manual approaches (8 reports), and neuromodulation, cueing, biofeedback, or other physical interventions (20 reports). These are counts of reports, not necessarily independent trial cohorts. The Ni et al. reports (11, 12) were treated as one trial family, as were the Lei et al. and Toosizadeh et al. reports (13, 14). Sex distribution and motor phenotype were variably reported in the source literature; the tables now show these fields consistently and use NR when a source did not provide a value that could be verified.

Motor endpoints most often concerned global motor severity, gait, balance, mobility, freezing of gait, falls, tremor, or hypomimia. Non-motor outcomes such as constipation, dysphagia, cognition, emotion recognition, biomarkers, mood, and quality of life were excluded from the motor synthesis unless they were secondary measures in an otherwise eligible motor-focused report. Most studies were small and short, and adverse-event reporting was inconsistent across intervention classes.

### 3.3 Evidence map of randomized reports

Tables 1–4 present published reports as the unit of display. The independent-trial count is lower because two report pairs arise from the same underlying randomized cohorts. “NR” is used when sex distribution or safety information could not be verified from the source report.

**Table 1:** Rehabilitation and technology-assisted training reports.

Study/Report	*N*	Sex	Motor phenotype/Target	Intervention	Comparator	Duration	Mapped motor outcome(s)	Safety/Adverse events
Goffredo et al. (15)	97	NR	Postural stability/mobility	Non-immersive VR telerehabilitation	Usual home rehabilitation	6–10 weeks	Mini-BESTest; TUG; 6MWT; MDS-UPDRS III	NR
Seçkinoğulları et al. (16)	28	NR	Axial mobility/balance/gait	Lumbosacral mobilization	No intervention	Single 10-min session	UPDRS; MPAS; DGI; posturography	NR
Carpinella et al. (17)	37	NR	Balance/gait	Wearable sensor/gamepad-based tailored training	Structured physical therapy	NR	BBS; 10MWT; stability index	NR
Gandolfi et al. (18)	76	NR	Postural instability	Home-based VR telerehabilitation	Sensory-integration balance training	7 weeks	BBS; 10MWT; ABC	NR
Hajebrahimi et al. (19)	24	NR	Global motor function/balance	VR exergaming	Exercise therapy	4 weeks	UPDRS-III; BBS; TUG; 6MWT	NR
Santos et al. (20)	41	NR	Balance/mobility	Nintendo Wii plus conventional exercise	Conventional exercise	8 weeks	BBS; TUG; DGI	NR
Ricciardi et al. (21)	36	NR	Hypomimia/facial motor function	DVD-guided facial rehabilitation	No treatment	8 weeks	UPDRS-III; facial motor outcomes	NR

ABC, Activities-specific Balance Confidence Scale; BBS, Berg Balance Scale; DGI, Dynamic Gait Index; MDS-UPDRS III, Movement Disorder Society–Unified Parkinson’s Disease Rating Scale Part III; Mini-BESTest, Mini-Balance Evaluation Systems Test; MPAS, Modified Parkinson’s Activity Scale; NR, not reported; PD, Parkinson’s disease; TUG, Timed Up and Go; UPDRS, Unified Parkinson’s Disease Rating Scale; VR, virtual reality; 6MWT, 6-min walk test; 10MWT, 10-m walk test.

**Table 2:** Mind–body and exercise-based reports.

Study/Report	*N*	Sex	Motor phenotype/Target	Intervention	Comparator	Duration	Mapped motor outcome(s)	Safety/Adverse events
Chang et al. (22)	43	NR	Global motor function	Aerobic exercise; Tai Chi Chuan	Usual activity	12 weeks	UPDRS-III	NR
Lee et al. (23)	41	NR	Global motor function/balance	Turo/Qi Dance	No additional treatment	8 weeks	UPDRS-III; BBS	NR
Rios Romenets et al. (24)	33	NR	Motor function/balance	Argentine tango	Self-directed exercise	12 weeks	UPDRS-III; Mini-BESTest	NR
Ni et al.^†^ (11)	41	NR	Global motor/lower-limb performance	Power training and high-speed yoga	No exercise	12 weeks	UPDRS-III; BBS; TUG; 10MWT	NR
Xiao and Zhuang (25)	96	NR	Gait/balance/global motor	Baduanjin plus walking	Walking only	6 months	UPDRS-III; 6MWT; BBS; TUG; gait speed	NR
Zhu et al. (26)	41	NR	Global motor/balance	Simplified Tai Chi plus usual exercise	Usual exercise	12 weeks	UPDRS-III; BBS	NR
Cherup et al. (27)	33	NR	Balance/proprioception	Yoga meditation	Proprioceptive training	12 weeks	FES; BESS; TUG	NR
Hulbert et al. (28)	24	NR	Turning/coordination	Dance class	Usual care	10 weeks	Turning kinematics; SS180	NR
Kunkel et al. (29)	51	NR	Mobility/balance	Partnered ballroom dance	Usual care	10 weeks	SS180; TUG; ABC; BBS; 6MWT	NR
Kurt et al. (30)	40	NR	Balance/global motor	Ai Chi exercise	Land exercise	5 weeks	BBS; TUG; UPDRS-III	NR
Van Puymbroeck et al. (31)	27	NR	Balance/gait/FOG	Yoga	No yoga	8 weeks	MDS-UPDRS; Mini-BESTest; FGA; FOG measures	NR
Shanahan et al. (32)	90	NR	Gait/balance/global motor	Irish set dancing plus usual care	Usual care	10 weeks	UPDRS-III; 6MWT; Mini-BESTest	NR
Ni et al.^†^ (12)	27 (secondary report)	NR	Global motor function	Power yoga	Usual care/non-exercise comparator	12 weeks	UPDRS; Hoehn and Yahr	NR
Li et al. (33)	40	NR	Gait/balance/global motor	Wuqinxi Qigong	Stretching	12 weeks	Gait parameters; MDS-UPDRS; TUG; Mini-BESTest	NR
Li et al. (34)	95	NR	Gait/balance/global motor	Tai Chi; brisk walking	No exercise	12 months	BBS; UPDRS; TUG	NR

ABC, Activities-specific Balance Confidence Scale; BBS, Berg Balance Scale; BESS, Balance Error Scoring System; FES, Falls Efficacy Scale; FGA, Functional Gait Assessment; FOG, freezing of gait; MDS-UPDRS, Movement Disorder Society–Unified Parkinson’s Disease Rating Scale; Mini-BESTest, Mini-Balance Evaluation Systems Test; NR, not reported; PD, Parkinson’s disease; SS180, standing-start 180-degree turn test; TUG, Timed Up and Go; UPDRS, Unified Parkinson’s Disease Rating Scale; 6MWT, 6-min walk test; 10MWT, 10-m walk test. ^†^The two Ni et al. reports are treated as publications from the same randomized trial family and count as one independent trial.

**Table 3:** Acupuncture-related and manual reports.

Study/Report	*N*	Sex	Motor phenotype/Target	Intervention	Comparator	Duration	Mapped motor outcome(s)	Safety/Adverse events
Cho et al. (36)	63	NR	Global motor function	Acupuncture plus bee venom acupuncture	Sham acupuncture/saline plus usual care	12 weeks	UPDRS-III; UPDRS-II	NR
Li et al. (37)	166	NR	Global motor function	Electroacupuncture	Usual care	12 weeks	UPDRS-III; prespecified motor outcomes	NR
Brandín-de la Cruz et al. (38)	33 analyzed	39% female	Gait/muscle tone	Dry needling	Sham dry needling	Single session; 7-day follow-up	TUG; 10MWT; 6MWT; myotonometry	NR
Hong et al. (35)	52	NR	Global motor function/mobility	Jiao’s scalp acupuncture plus VR rehabilitation	VR rehabilitation	8 weeks	TUG; UPDRS-III	NR
Jang et al. (39)	26	NR	Gait/global motor function	Acupuncture plus usual care	Usual care	4 weeks	10MWT; TUG; UPDRS-III	NR
Lei et al.^‡^ (13)	15	NR	Gait	Electroacupuncture/acupoint stimulation	Sham acupoints	3 weeks	Gait speed; UPDRS-III	NR
Toosizadeh et al.^‡^ (14)	15	NR	Balance/global motor function	Electroacupuncture	Sham stimulation	3 weeks	Short FES-I; UPDRS	Transient lightheadedness reported in the linked trial family; no serious AE reported
Pereira et al. (40)	7	NR	Balance/gait	Acupuncture	Sham acupuncture	3 months	Balance and gait parameters	NR

AE, adverse event; FES-I, Falls Efficacy Scale–International; MDS-UPDRS, Movement Disorder Society–Unified Parkinson’s Disease Rating Scale; NR, not reported; TUG, Timed Up and Go; UPDRS, Unified Parkinson’s Disease Rating Scale; VR, virtual reality; 6MWT, 6-min walk test; 10MWT, 10-meter walk test. ^‡^Lei et al. and Toosizadeh et al. are linked reports from the same 15-participant randomized trial family and count as one independent trial.

**Table 4:** Neuromodulation, cueing, biofeedback, and other physical-intervention reports.

Study/Report	*N*	Sex	Motor phenotype/Target	Intervention	Comparator	Duration	Mapped motor outcome(s)	Safety/Adverse events
Del Felice et al. (41)	15	NR	Global motor function	Personalized tACS plus physical therapy	Random-noise stimulation	2 weeks	UPDRS-III	NR
Romero et al. (42)	40	NR	Global motor/balance	rTMS, EEG-guided neurofeedback, or combination	No treatment	2 weeks	UPDRS-III; TUG; limits of stability	NR
Zhang et al. (43)	13	NR	Tremor	Bilateral tDCS	Sham tDCS	Single 20-min session	UPDRS-III; tremor scales	NR
Yang et al. (44)	12	NR	Global motor function	Transcranial temporal interference stimulation	Sham stimulation	2 weeks	UPDRS-III	NR
Pagnussat et al. (45)	33 PD randomized	NR	Gait/functional mobility	Automated mechanical peripheral stimulation	Sham stimulation	8 sessions	TUG; gait parameters	NR
Li et al. (46)	32 randomized	NR	Global motor/balance	Whole-body vibration	Conventional therapy	Single 10-min treatment	UPDRS motor; FRT; TUG	No serious safety signal highlighted; detailed AE reporting limited
Roßkopf et al. (47)	34	NR	Hypomimia/facial motor function	Facial EMG biofeedback	No facial exercise	NR	Facial motor-expression measures	NR
Braun Janzen et al. (48)	37	NR	Upper-limb motor timing/global motor	Finger-tapping/arm-swing rhythmic priming	No training	NR	UPDRS-III/IV; BBS; NHPT	NR
Calabrò et al. (49)	50	NR	Gait/mobility	Rhythmic auditory stimulation with treadmill/music	Treadmill without RAS	8 weeks	FGA; 10MWT; UPDRS; BBS; TUG	NR
Hubble et al. (50)	22	NR	Balance	Trunk-specific exercise	Education	12 weeks	Static balance; TUG; UPDRS-III	NR
Hutin et al. (51)	15	NR	Gait	Adaptive rhythmic/spatial auditory cueing	Crossover control condition	Single session	10-m walking speed	NR
Yakşi et al. (52)	41	NR	Balance/mobility	Static posturography-assisted biofeedback plus balance training	Usual balance training	6 weeks	MDS-UPDRS; BBS; TUG; TST	NR
Thaut et al. (53)	61	NR	Falls/balance	Rhythmic auditory stimulation	Withdrawal/control phase	24 weeks	Falls efficacy; BBS	NR
Pohl et al. (54)	46 randomized	NR	Dual-task mobility/balance/FOG	Group-based music intervention	No music training	12 weeks	Dual-task TUG; balance; FOG measures	NR
Mitarnun et al. (55)	33	NR	Gait/mobility	Walking meditation	Usual care	12 weeks	TUG; gait speed; FTSTS	NR
Bezerra et al. (56)	39	NR	Balance/FOG	Action observation + motor imagery + gait training	Educational video + gait sessions	4 weeks	Mini-BESTest; FOG-Q	NR
Abraham et al. (57)	20	NR	Gait/functional mobility	Dynamic neuro-cognitive imagery training	Practice control	16 h total	TUG; 6MWT; chair stand; gait speed	NR
Tinaz et al. (58)	44	NR	Global motor function	Neurofeedback-guided kinesthetic motor imagery	Active visual-imagery control	4 weeks	UPDRS-III	NR
DiFrancisco-Donoghue et al. (59)	11 randomized; 9 completed	NR	Global motor/balance	Osteopathic manipulative medicine	Counseling	6 weeks	Mini-BESTest; MDS-UPDRS; SOT	NR
Miyahara et al. (60)	90	45 F/45 M	ON-freezing of gait/gait	Therapeutic Thai acupressure (TTA) or laser cane (LC)	Sham light-touch control; 3-arm trial (30/arm)	Single-session immediate-effect protocol	Stride length; FOG; TUG; gait parameters	No adverse events reported

AE, adverse event; BBS, Berg Balance Scale; EEG, electroencephalography; EMG, electromyography; FGA, Functional Gait Assessment; FOG, freezing of gait; FOG-Q, Freezing of Gait Questionnaire; FRT, Functional Reach Test; FTSTS, Five Times Sit-to-Stand; LC, laser cane; MDS-UPDRS, Movement Disorder Society–Unified Parkinson’s Disease Rating Scale; NHPT, Nine-Hole Peg Test; NR, not reported; PD, Parkinson’s disease; RAS, rhythmic auditory stimulation; rTMS, repetitive transcranial magnetic stimulation; SC, sham control; SOT, Sensory Organization Test; tACS, transcranial alternating current stimulation; tDCS, transcranial direct current stimulation; TTA, therapeutic Thai acupressure; TST, tandem stance test; TUG, Timed Up and Go; UPDRS, Unified Parkinson’s Disease Rating Scale; VAS, visual analogue scale; 6MWT, 6-min walk test; 10MWT, 10-meter walk test. Non-motor secondary outcomes were not used to characterize motor effects.

### 3.4 Rehabilitation and technology-assisted training

Seven reports evaluated rehabilitation or technology-assisted training (15–21). These interventions included telerehabilitation, virtual-reality training, interactive sensor/game-based approaches, a single-session lumbosacral mobilization study, conventional exercise combined with gaming, and facial rehabilitation. Importantly, these interventions are described here as rehabilitation rather than as complementary or alternative medicine. The Seçkinoğulları et al. study (16) tested the acute effect of one 10-min lumbosacral mobilization session, not a 6-week intervention. Across this category, studies mapped postural stability, balance, gait, mobility, global motor scores, or hypomimia; heterogeneity in comparator intensity and treatment dose precludes a common efficacy interpretation.

### 3.5 Mind–body and exercise-based interventions

Fifteen eligible reports described Tai Chi, Qigong/Wuqinxi/Baduanjin, yoga, dance-based training, Ai Chi, and related movement programs (11, 12, 22–34). One previously included dance report was removed because it used a quasi-randomized design rather than true random allocation. The two Ni et al. publications (11, 12) were linked as reports from the same study family and are not counted as separate independent trials. Intervention duration ranged from weeks to 12 months, and motor endpoints included global motor severity, gait, balance, turning, lower-limb performance, and freezing of gait. Because active comparators and intervention content differed considerably, this map should not be read as ranking one movement practice over another.

### 3.6 Acupuncture-related and manual approaches

Eight eligible reports evaluated acupuncture-related or manual interventions (13, 14, 35–40). The scalp-acupuncture-plus-VR report (35) is listed only in this category, eliminating the duplicate table entry present in the previous version. Two electroacupuncture papers (13, 14) were identified as reports from the same 15-participant trial family and are linked accordingly. A dysphagia-only acupuncture report was removed because swallowing outcomes did not meet the prespecified motor-function eligibility criterion for this review. Dry needling was evaluated as a single treatment session with 7-day follow-up, and the report found no between-group advantage for the prespecified gait outcomes (38). Acupuncture point selection, stimulation parameters, comparators, and treatment dose remained highly variable across reports.

### 3.7 Neuromodulation, cueing, biofeedback, and other physical interventions

Twenty reports evaluated non-invasive brain stimulation, auditory or visual cueing, biofeedback, mechanical peripheral stimulation, whole-body vibration, trunk exercise, motor imagery, walking meditation, osteopathic manipulation, and related approaches (41–60). A conduction-exercise/self-acupressure report was removed because the extractable outcomes in the report were non-motor or quality-of-life measures rather than eligible motor endpoints. Several data-extraction errors in the prior table were corrected. Pagnussat et al. randomized 33 participants with PD to active or sham peripheral stimulation and included a separate healthy reference group; the randomized PD sample is therefore reported as 33, not 44 (45). Li et al. randomized 32 participants to whole-body vibration or conventional therapy (46). Pohl et al. randomized 46 participants, not 50 (54). DiFrancisco-Donoghue et al. randomized 11 participants, of whom nine completed the study (59). Miyahara et al. was a three-arm, single-session trial (30 participants each to therapeutic Thai acupressure, laser-cane cueing, or sham light touch), rather than a two-group comparison with “standard treatment” (60).

## 4 Discussion

### 4.1 Principal findings

After reconciling duplicate table entries, eligibility, and publication families, this scoping review contains 50 eligible reports representing 48 independent randomized trials. The evidence spans conventional rehabilitation, complementary/integrative movement practices, acupuncture/manual approaches, and other adjunctive technologies. This breadth is useful for mapping where randomized research exists, but it also explains why the evidence cannot be summarized as a single treatment class or a single effect. Motor outcomes were most often global motor scores, gait, balance, mobility, freezing of gait, falls, tremor, or hypomimia; treatment dose, comparator design, follow-up, and safety reporting were inconsistent.

### 4.2 Conceptual boundary of complementary, integrative, and rehabilitative care

The previous manuscript used CATs as an umbrella for virtually all non-pharmacological interventions. That wording obscured an important distinction. Complementary care is defined by its relationship to conventional medicine rather than by the mere absence of a drug or operation (6). Conventional physiotherapy, task-specific rehabilitation, and telerehabilitation are established rehabilitation approaches, whereas acupuncture, Tai Chi, Qigong, yoga, and some manual practices are commonly framed as complementary/integrative in the PD literature. Cueing, biofeedback, VR platforms, and non-invasive stimulation occupy yet another category of adjunctive technologies. The revised terminology is therefore intentionally functional: ANPI is the overarching evidence-mapping category, with complementary/integrative interventions treated as one subgroup rather than the definition of the entire evidence base.

### 4.3 Methodological concerns and evidence gaps

Several limitations recur across the mapped literature. Samples were frequently small and follow-up was often short. Motor phenotype was not consistently used as an eligibility or stratification variable, even though tremor, freezing of gait, axial impairment, postural instability, and bradykinesia may require different outcome constructs. Outcome selection was also fragmented; UPDRS-III/MDS-UPDRS III was common, but gait, balance, falls, turning, mobility, and phenotype-specific measures varied widely. Reporting of adherence, co-interventions, treatment fidelity, and adverse events was incomplete. Multiple publications from the same cohort can also inflate an evidence map if reports are mistaken for independent trials; the revised analysis addresses this explicitly for two identified study families.

Future randomized studies should prespecify the motor phenotype and the primary motor endpoint, state medication ON/OFF conditions where relevant, report intervention dose and progression in reproducible detail, and include follow-up that is long enough to distinguish an immediate performance effect from a sustained training effect. Adverse events and adherence should be reported even when no events occur. For future systematic reviews intended to support comparative effectiveness claims, formal risk-of-bias assessment, extraction of between-group effect estimates, trial-family linkage, and certainty-of-evidence methods will be necessary.

### 4.4 Interpretation of the mapped findings

Because this review did not perform a formal risk-of-bias assessment, did not pool between-group effect estimates, and did not systematically grade safety evidence, it cannot determine which intervention is effective, superior, or appropriate for an individual patient. Apparent positive findings in individual reports should be interpreted as study-level signals within a heterogeneous evidence base rather than as practice recommendations. The revised manuscript therefore removes phenotype-specific treatment advice and the previous statement that the data “support individualized adjunctive use.”

### 4.5 Strengths and limitations of this review

The main strength of this revision is the clearer separation of intervention categories and the explicit distinction between published reports and independent randomized trials. The evidence tables now present randomized sample size, sex distribution when verifiable, motor target, motor outcomes, and safety reporting in a consistent format, and linked publications are flagged. The revision also removes non-motor-only reports and corrects several source-level extraction and citation errors.

Important limitations remain. First, the OSF registration did not prospectively prespecify the final motor-symptom review: the original focus was non-motor symptoms, and the final study-design and database plan also deviated from the registration. This post-registration change increases the possibility of selective methodological decisions and should be considered when interpreting the review. Second, the search was limited to PubMed and Web of Science, and only English- and Chinese-language full texts were eligible; relevant studies indexed elsewhere or in other languages may have been missed. Third, no formal risk-of-bias assessment or meta-analysis was performed. The present work should therefore be read strictly as a descriptive map of randomized reports, not as evidence of comparative efficacy, safety, or clinical effectiveness.

## 5 Conclusion

Randomized research on adjunctive non-pharmacological interventions for PD motor symptoms is extensive in scope but heterogeneous in design and reporting. In this revised map, 50 eligible reports represent 48 independent trials across rehabilitation, complementary/integrative movement practices, acupuncture/manual approaches, and other adjunctive technologies. The available literature identifies frequently studied motor targets—particularly gait, balance, mobility, freezing of gait, and global motor severity—but does not, in the absence of formal risk-of-bias assessment and comparative effect synthesis, establish treatment efficacy or support clinical recommendations. More reproducible trials and reviews should prespecify motor phenotypes, link multiple reports from the same trial, use harmonized motor outcomes, and report safety and adherence systematically.

## Data Availability

The datasets presented in this study can be found in online repositories. The names of the repository/repositories and accession number(s) can be found in the article/ Supplementary material.

## Footnotes

1
https://osf.io/tu5nd/
