# Supplementary material for: Adjunctive non-pharmacological interventions for motor symptoms in Parkinson’s disease: a scoping review of randomized controlled trials
Source: Front Neurol. 2026 Sep 18;17:1926421. doi: 10.3389/fneur.2026.1926421 (PMC13630624; doi:10.3389/fneur.2026.1926421)
Supplement: Supplementary file 1 [file Data_Sheet_1.pdf]

Supplementary Material

Adjunctive non-pharmacological interventions for motor symptoms in Parkinson's disease: a scoping review of randomized controlled trials

Ziyuan Wang, Jie Ruan, Mengjiao Zhang, and Chen Li

Supplementary Appendix A. Complete search strategies

PubMed (run 7 September 2025)

```
(("Parkinson Disease"[Mesh] OR "Parkinson disease"[tiab] OR "Parkinson's disease"[tiab] OR
parkinsonism[tiab]) AND ("Complementary Therapies"[Mesh] OR complementary therap*[tiab] OR
alternative therap*[tiab] OR integrative medicine[tiab] OR rehabilitat*[tiab] OR exercise[tiab]
OR "exercise therapy"[tiab] OR "Tai Chi"[tiab] OR qigong[tiab] OR Wuqinxi[tiab] OR
Baduanjin[tiab] OR yoga[tiab] OR dance[tiab] OR acupuncture[tiab] OR electroacupuncture[tiab]
OR acupressure[tiab] OR "dry needling"[tiab] OR "virtual reality"[tiab] OR
telerehabilitation[tiab] OR cueing[tiab] OR "rhythmic auditory stimulation"[tiab] OR
biofeedback[tiab] OR neuromodulation[tiab] OR "transcranial stimulation"[tiab] OR "motor
imagery"[tiab] OR "whole-body vibration"[tiab] OR "whole body vibration"[tiab] OR
osteopathic[tiab]) AND (randomized controlled trial[pt] OR controlled clinical trial[pt] OR
random*[tiab] OR trial[tiab])) AND ("2015/01/01"[Date - Publication] : "2025/09/07"[Date -
Publication]) AND (english[lang] OR chinese[lang])
```

Web of Science Core Collection (run 7 September 2025)

```
TS=(("Parkinson disease" OR "Parkinson's disease" OR parkinsonism) AND ("complementary therap*"
OR "alternative therap*" OR "integrative medicine" OR rehabilitat* OR exercise OR "exercise
therapy" OR "Tai Chi" OR qigong OR Wuqinxi OR Baduanjin OR yoga OR dance OR acupuncture OR
electroacupuncture OR acupressure OR "dry needling" OR "virtual reality" OR telerehabilitation
OR cueing OR "rhythmic auditory stimulation" OR biofeedback OR neuromodulation OR "transcranial
stimulation" OR "motor imagery" OR "whole-body vibration" OR "whole body vibration" OR
osteopathic) AND (random* OR trial))
```

Refinements: publication years 2015-2025; document type Article; languages English OR Chinese; records published after 7 September 2025 were excluded during export/screening.

Search-count note: The flow diagram reports the numbers exported on the stated search date. Because database indexing changes over time, a rerun on a later date may not return an identical count even when the strategy is unchanged.

Supplementary Appendix B. Protocol deviations and clarifications

Domain	Registered / previous plan	Implemented final review	Status
**Review focus/title**	Registered OSF project initially focused on non-motor symptoms of PD.	Final review focuses on motor symptoms and motor function.	Post-search substantive change; disclosed in Methods and limitations.
**Study-design scope**	Registered plan differed from the final design scope.	Restricted to randomized controlled, randomized crossover, and randomized pilot/feasibility trials; quasi-randomized studies excluded.	Post-registration deviation.
**Information sources**	Registered database plan differed from the implemented review.	PubMed and Web of Science Core Collection used in the final review.	Post-registration deviation.
**Language criterion**	Previous manuscript wording was operationally ambiguous.	English- or Chinese-language full-text reports eligible; other languages excluded.	Clarified for reproducibility.
**Unit of counting**	Published reports and independent trials were not clearly separated.	Both report count and independent-trial count are now reported; linked reports are flagged.	Added in response to peer review.
**Outcome handling**	Previous tables mixed motor and non-motor outcomes.	At least one eligible motor/motor-function endpoint required; non-motor-only reports excluded; secondary non-motor outcomes not synthesized.	Clarified in revision.
